# Involvement of A_1_ adenosine receptors in osmotic volume regulation of retinal glial cells in mice

**Published:** 2009-09-12

**Authors:** Antje Wurm, Stephan Lipp, Thomas Pannicke, Regina Linnertz, Katrin Färber, Peter Wiedemann, Andreas Reichenbach, Andreas Bringmann

**Affiliations:** 1 Paul Flechsig Institute of Brain Research, University of Leipzig, Leipzig, Germany; 2 Department of Ophthalmology and Eye Hospital, University of Leipzig, Leipzig, Germany; 3 Cellular Neurosciences, Max-Delbrück-Center for Molecular Medicine, Berlin, Germany

## Abstract

**Purpose:**

Osmotic swelling of Müller glial cells has been suggested to contribute to retinal edema. We determined the role of adenosine signaling in the inhibition of Müller cell swelling in the murine retina.

**Methods:**

The size of Müller cell somata was recorded before and during perfusion of retinal sections and isolated Müller cells with a hypoosmolar solution. Retinal tissues were freshly isolated from wild-type mice and mice deficient in A_1_ adenosine receptors (*A_1_AR^−/−^*), or cultured as whole-mounts for three days. The potassium conductance of Müller cells was recorded in isolated cells, and retinal slices were immunostained against Kir4.1.

**Results:**

Hypotonic exposure for 4 min induced a swelling of Müller cell bodies in retinal slices from *A_1_AR^−/−^* mice but not wild-type mice. Pharmacological inhibition of A_1_ receptors or of the ecto-5′-nucleotidase induced hypoosmotic swelling of Müller cells from wild-type mice. Exogenous adenosine prevented the swelling of Müller cells from wild-type but not *A_1_AR^−/−^* mice. The antiinflammatory corticosteroid, triamcinolone acetonide, inhibited the swelling of Müller cells from wild-type mice; this effect was blocked by an antagonist of A_1_ receptors. The potassium conductance of Müller cells and the Kir4.1 immunolabeling of retinal slices were not different between *A_1_AR^−/−^* and wild-type mice, both in freshly isolated tissues and retinal organ cultures.

**Conclusions:**

The data suggest that autocrine activation of A_1_ receptors by extracellularly generated adenosine mediates the volume homeostasis of Müller cells in the murine retina. The swelling-inhibitory effect of triamcinolone is mediated by enhancement of endogenous adenosine signaling.

## Introduction

The development of retinal edema is an important complication of various ocular diseases such as diabetic retinopathy and uveitis [1-3]. The mechanisms of retinal edema formation are incompletely understood. It has been shown that retinal ischemia, inflammation, and oxidative stress are causative factors of edema [2,3]. Generally, there are two basic mechanisms of water accumulation in neural tissues: vasogenic edema, characterized by a breakdown of the blood-neural tissue barrier and vascular leakage, and cytotoxic edema caused by intracellular water accumulation resulting in cellular swelling [4]. Both mechanisms were suggested to contribute to the development of retinal edema in the human tissue [2,3,5,6] and in animal studies of retinopathies [7-9].

Removal of extraneous fluid in retinal edema aids in restoration of vision [10]. The antiinflammatory corticosteroid, triamcinolone acetonide (9α-fluoro-16α-hydroxyprednisolone), is commonly used to treat retinal edema [11,12]. Triamcinolone decreases the blood-ocular barrier breakdown [13], apparently through multiple mechanisms including a decrease in the level of the major vasopermeabilizing factor, vascular endothelial growth factor [14]. Triamcinolone acetonide resolves macular edema also in patients that do not display angiographic vascular leakage. This suggests that triamcinolone may also reduce cytotoxic edema, i.e., swelling of retinal cells. In animal models, the edema-resolving effect of triamcinolone was suggested to be mediated by inhibition of both vasogenic and cytotoxic edema. Triamcinolone reduces vascular leakage [15,16] and suppresses the leukocyte-endothelial interaction [16These effects were mediated by a decrease in the secretion of vascular endothelial growth factor from retinal cells [17,18] and by inhibition of the activation of metalloproteinases [18,19]. Triamcinolone was shown to prevent the osmotic swelling of Müller glial cells in tissue preparations of the rat retina [20]. Osmotic swelling is a characteristic feature of Müller cells in animal models of retinal ischemia, detachment, ocular inflammation, and diabetes [21-24]. It has been suggested that the inhibitory action of triamcinolone on the swelling of Müller cells is mediated by adenosine and activation of adenosine A_1_ receptors [20]. The previous data suggest that A_1_ receptor signaling may play an important role in preventing the osmotic swelling of Müller cells under pathological conditions [20]. However, it is unclear whether endogenous adenosine may have cell volume-regulatory effects also in the healthy retina. Therefore, we investigated the role of adenosine signaling in the regulation of the Müller cell volume in the murine retina, and compared the osmotic swelling characteristics of Müller cells in retinal tissues from wild-type mice and mice deficient in A_1_ adenosine receptors (*A_1_AR^–/–^*). In addition, we investigated short-term organ cultures of retinal tissues from both animal strains. It has been shown recently that retinal organ culturing is associated with an alteration in the swelling characteristics of Müller cells similar to ischemic and inflammatory conditions in vivo [25]. Induction of osmotic swelling was shown to be associated with a decrease in the potassium conductance of Müller cells in rats [21-23]. Therefore, we determined also the potassium currents and the distribution of the major glial potassium channel, Kir4.1, in Müller cells and retinal tissues from wild-type and *A_1_AR^–/–^* mice.

## Methods

### Materials

Papain was purchased from Roche Molecular Biochemicals (Mannheim, Germany). Chloromethyltetramethylrosamine (Mitotracker Orange) was from Molecular Probes (Eugene, OR). DNase I, adenosine-5′-O-(α,β-methylene)-diphosphate (AOPCP), bis-(o-aminophenoxy) ethane-*N*,*N*,*N'*,*N'*-tetra-acetic acid acetoxymethyl ester (BAPTA/AM), 8-cyclopentyl-1,3-dipropylxanthine (DPCPX), 3-isobutyl-1-methylxanthine (IBMX), 5-nitro-2-(3-phenylpropylamino) benzoic acid (NPPB), 8-(4-chlorophenylthio)-cyclic AMP (pCPT-cAMP), and all other substances used were purchased from Sigma-Aldrich (Taufkirchen, Germany), unless stated otherwise. The following antibodies were used: 1:200 rabbit anti-Kir4.1 (Sigma), 1:200 mouse anti-glial fibrillary acidic protein (GFAP; G-A-5 clone, Sigma), 1:400 Cy3-conjugated goat anti-rabbit IgG (Dianova, Hamburg, Germany), and 1:400 Cy2-coupled goat anti-mouse IgG (Dianova).

### Animals

All experiments were done in accordance with the European Communities Council Directive 86/609/EEC, and were approved by the local authorities (Faculty of Medicine of the University of Leipzig and Regierungspräsidium Leipzig). Animals were adult (2–6 months) homozygous A_1_AR null mice and wild-type controls (both in a 129Sv/C57BL/6 background). Offspring from intercrosses were genotyped by PCR analysis as described [26]. For genotyping, tail DNA was isolated and tested for the presence of wild-type and mutant genes, with A1AR and (Neo-R) specific primers generating PCR products of 444 bp from the wild-type allele and 457 bp from the mutant allele. Primer sequences were sense A1AR, 5′-GTA CAT CTC GGC CTT CCA GG-3′; antisense A1AR, 5′-GAG AAT ACC TGG CTG ACT AG-3′; sense neo-R, 5′-ACA ACA GAC AAT CGG CTG CTC TGA TG-3′; and antisense neo-R, 5′-TGC GCG CCT TGA GCC TGG CGA AC-3′. Animals were maintained with free access to water and food in an air-conditioned room on a 12 h:12 h light-dark cycle, and were sacrificed with carbon dioxide.

### Preparation of retinal slices

Pieces of freshly isolated retinas (5×5 mm) were placed, with the photoreceptor side down, onto membrane filters (mixed cellulose ester, 0.45 µm pore size; Schleicher & Schuell MicroScience, Dassel, Germany). Retinal slices of 1 mm thickness were cut from these tissues adhering to the membrane filters, using a custom-made cutter equipped with a razor blade.

### Preparation of isolated Müller cells

Pieces of isolated retinas were incubated in papain (0.2 mg/ml)-containing calcium-free and magnesium-free phosphate-buffered saline, pH 7.4, for 30 min at 37 °C. The phosphate-buffered saline contained 8 g/l NaCl, 0,2 g/l KCl, 1,15 g/l Na_2_HPO_4_, and 0,2 g/l KH_2_P0_4_. After three washing steps (1 min each) in phosphate-buffered saline containing calcium chloride (2 mM) and magnesium chloride (1 mM), the tissue pieces were incubated in calcium-containing and magnesium-containing phosphate-buffered saline supplemented with DNase I (200 U/ml) for 2 min. Thereafter, the tissue pieces were triturated by a wide-pore pipette, to obtain isolated Müller cells. The cells were stored at 4 °C in serum-free minimum essential medium until use within 1.5 h after cell isolation.

### Short-term retinal organ cultures

The retinas were isolated and were cut into two equal pieces. Each piece was whole-mounted onto a membrane filter with the vitreal side up. The membrane filters were put on sterilized high-grade steel grids, which were placed in a Petri dish filled with culture medium up to a level just below the upper vitreal surface of the explant. The culture medium consisted of 10.64 g/l minimal essential medium, 50 mg/l gentamycin, 2.766 g/l HEPES, 2.1 g/l NaHCO_3_, and 10% fetal calf serum resulting in a pH of 7.4 in an incubator with an atmosphere containing 5% CO_2_ and a temperature of 37 °C. After three days in vitro, isolated Müller cells or retinal tissues were investigated. Data were obtained in two independent cultures; two wild-type and *A_1_AR^–/–^* animals were used for each culture, resulting in a total number of four mice of each genotype used for the in vitro experiments.

### Müller cell swelling

All experiments were performed at room temperature (20–23 °C). To monitor volume changes of Müller cells in response to hypotonic challenge, the somata of Müller cells in the inner nuclear layer of retinal slices, or of freshly isolated Müller cells, were focused. The filter stripes with the retinal slices were transferred to recording chambers and kept submerged in extracellular solution by a metal grid with nylon threads. The chambers were mounted on the stage of an upright confocal laser scanning microscope (LSM 510 Meta; Zeiss, Oberkochen, Germany). Retinal slices and isolated cells were loaded with 10 µM of the vital dye Mitotracker Orange; it has been shown that this dye stains selectively the somata of Müller glial cells in the inner nuclear layer of retinal tissues [27]. After incubation of the slices or cells with Mitotracker Orange-containing extracellular solution for three minutes, the slices or cells were perfused with extracellular solution at a flow rate of 2 ml/min, and recordings were made with an Achroplan 63x/0.9 water immersion objective. The pinhole was set at 172 µm; the thickness of the optical section was adjusted to 1 µm. Mitotracker Orange was excited at 543 nm with a HeNe laser, and emission was recorded with a 560 nm long-pass filter. Images were obtained with an x-y frame size of 256×256 pixel (73.1×73.1 µm). In the course of the experiments, the Mitotracker Orange-stained somata of Müller cells were recorded at the plane of their maximal extension. To insure that the maximum soma area was precisely measured, the focal plane was continuously adjusted in the course of the experiments.

### Solutions

A gravity-fed system with multiple reservoirs was used to perfuse the recording chamber continuously with extracellular solution; test substances were applied by rapidly changing the perfusate. The bathing solution in the recording chamber was totally changed within 2 min. The extracellular solution consisted of 136 mM NaCl, 3 mM KCl, 2 mM CaCl_2_, 1 mM MgCl_2_, 10 mM HEPES, and 11 mM glucose, adjusted to pH 7.4 with Tris. The hypoosmolar solution (60% of control osmolarity) was made up by adding distilled water. When barium-containing extracellular solutions were used, 1 mM barium chloride was added to iso- and hypoosmolar solutions, and the slices were preincubated in barium-containing isoosmolar extracellular solution for 10 min. Blocking substances were preincubated for 15 to 45 min, and agonists were applied simultaneously with the hypoosmolar solution.

### Whole-cell patch-clamp records of isolated Müller cells

The whole-cell currents of the cells were recorded at room temperature using the Axopatch 200A amplifier (Axon Instruments, Foster City, CA) and the ISO-2 computer program (MFK, Niedernhausen, Germany). The signals were low-pass filtered at 1, 2, or 6 kHz (eight-pole Bessel filter) and digitized at 5, 10, or 30 kHz, respectively, using a 12-bit A/D converter. Patch pipettes were pulled from borosilicate glass (Science Products, Frankfurt/Main, Germany) and had resistances between 4 and 6 MΩ when filled with a solution containing 10 mM NaCl, 130 mM KCl, 1 mM CaCl_2_, 2 mM MgCl_2_, 10 mM EGTA, and 10 mM HEPES, adjusted to pH 7.1 with Tris. The recording chamber was continuously perfused with extracellular solution that contained 135mM NaCl, 3 mM KCl, 2 mM CaCl_2_, 1 mM MgCl_2_, 1 mM Na_2_HPO_4_, 10 mM HEPES, and 11 mM glucose, equilibrated to pH 7.4 with Tris. Potassium currents were evoked by applying depolarizing and hyperpolarizing voltage steps of 250 ms duration, with increments of 10 mV, from a holding potential of −80 mV. The amplitude of the steady-state inward potassium (Kir) currents was measured at the end of the 250 ms voltage step from −80 to −140 mV. The membrane capacitance of the cells was measured by the integral of the uncompensated capacitive artifact (filtered at 6 kHz) evoked by a hyperpolarizing voltage step from −80 to −90 mV in the presence of 1 mM extracellular barium chloride. The resting membrane potential was measured in the current-clamp mode.

### Immunohistochemistry

Isolated retinas were fixed in 4% paraformaldehyde for 1 h. After several washing steps in buffered saline, the tissues were embedded in calcium-containing and magnesium-containing phosphate-buffered saline containing 3% agarose (w/v), and 70 µm thick slices were cut using a vibratome. The slices were incubated in 5% normal goat serum plus 0.3% Triton X-100 in calcium-containing and magnesium-containing phosphate-buffered saline for 2 h at room temperature and, subsequently, in the primary antibody overnight at 4 °C. After washing in 1% BSA, the secondary antibody was applied for 2 h at room temperature. Control slices were stained without the primary antibody; no nonspecific labeling was observed following incubation with the secondary antibody alone (not shown). Images were taken with the LSM 510 Meta (Zeiss).

### Data analysis

To determine the extent of the swelling of Müller cell somata, the cross-sectional area of Mitotracker Orange-stained cell bodies in the inner nuclear layer of retinal slices was measured off-line using the image analysis software of the laser scanning microscope. Bar diagrams display the mean (±SEM) cross-sectional areas of cell somata that were measured after a 4 min perfusion with the hypoosmolar solution, in percent of the soma area measured before hypotonic challenge. Statistical analysis was made by using the Prism program (Graphpad Software, San Diego, CA); significance was determined by the nonparametric Mann–Whitney *U* test.

## Results

### Osmotic swelling of Müller cells

**Figure 1:**
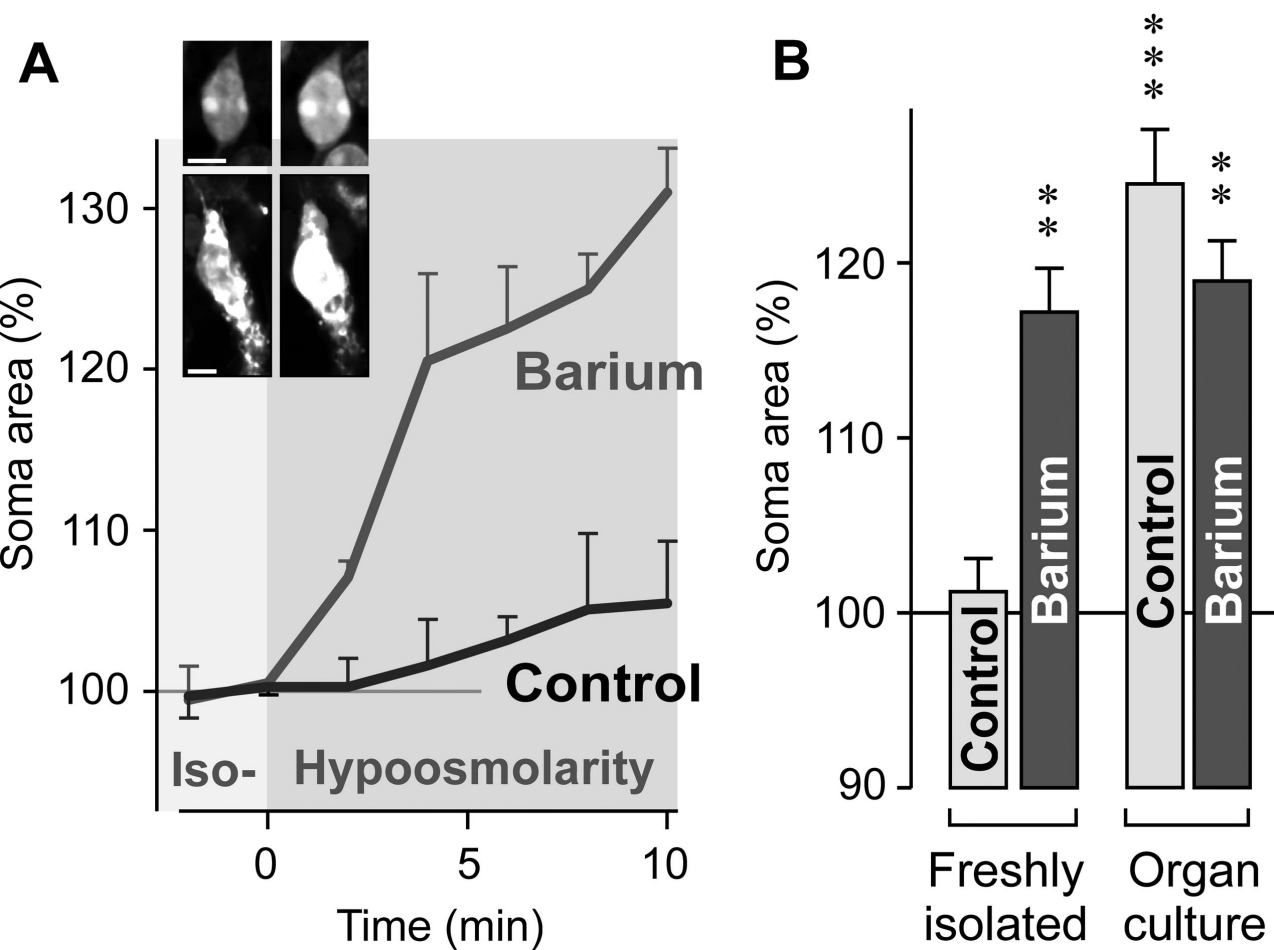
Osmotic swelling of Müller glial cells in slices of the wild-type mouse retina. **A**: Perfusion of slices of a freshly isolated retina with a hypoosmolar solution, containing 60% of control osmolarity, in the absence (control) and presence of 1 mM barium chloride resulted in a time-dependent swelling of Müller cell somata. The diagram displays the mean (±SEM) cross-sectional area of the somata (n=3 each). Inset images in **A** show original records of dye-filled Müller cell somata obtained before (left) and during (right) perfusion with the barium-containing hypoosmolar solution. Scale bars represents 5 µm. **B**: Hypoosmotic exposure for 4 min evoked swelling of Müller cell somata in slices of freshly isolated retinas in the presence but not absence of 1 mM barium chloride. Müller cells in slices of retinal organ cultures displayed soma swelling under both conditions. Data are mean (±SEM) soma areas (n=7–21 cells per bar) expressed in percent of the soma size recorded before hypoosmotic challenge (100%). Significant increases in the soma area: The double asterisk indicates a p<0.01 and the triple asterisk indicates a p<0.001.

The swelling of Müller cell somata was investigated by perfusion of freshly isolated retinal slices with a hypoosmolar solution (containing 60% of control osmolarity). Under isotonic conditions, the absolute soma areas of Müller cells from wild-type and knockout animals were not different (wild-type: 49.6±1.7 µm^2^, n=48; *A_1_AR^−/−^*: 45.8±1.3 µm^2^, n=60; p>0.05). Hypotonic exposure for 4 min did not evoke a significant swelling of glial cell bodies in freshly isolated retinal slices from wild-type mice (Figures 1A,B). The increase in the size of glial cell bodies was significantly (p<0.01) stronger and faster when 1 mM barium chloride was jointly applied with the hypoosmolar solution (Figures 1A,B). Barium ions are known to block the principal potassium conductance of Müller cells mediated by inwardly rectifying potassium (Kir) channels [28,29]. As shown recently, Müller cells in slices of retinal organ cultures of the rat display a decrease in their Kir currents which is associated with an induction of cellular swelling under hypotonic conditions [25]. We found that Müller cells in organ cultures of the wild-type mouse retina displayed a swelling of their cell bodies under hypoosmotic conditions in the absence and presence of barium ions (Figure 1B). The swelling in the absence of barium suggests that retina culturing alters the volume regulation mechanisms of Müller cells.

### Inhibition of Müller cell swelling by adenosine

**Figure 2:**
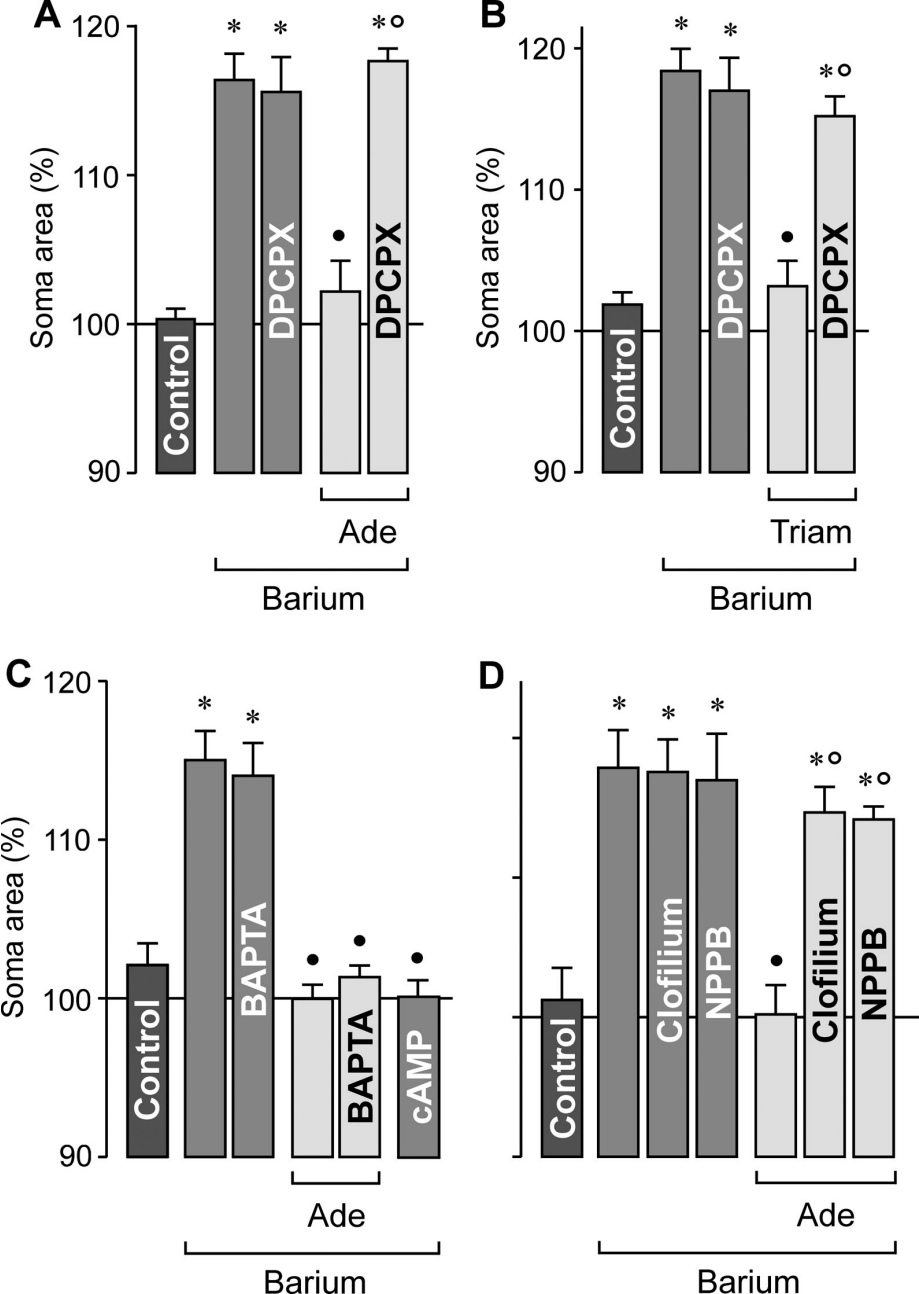
Adenosine inhibits the osmotic swelling of Müller cell somata in freshly isolated retinal slices from wild-type mice. The cross-sectional area of the somata was measured after a 4 min perfusion of the slices with a hypoosmolar solution (in the absence and presence of 1 mM barium chloride, as indicated), and are expressed in percent of the soma size measured before hypotonic challenge (100%). **A**: The swelling-inhibitory effect of 10 µM adenosine (Ade) was prevented in the presence of 100 nM DPCPX, a selective antagonist of adenosine A_1_ receptors. **B**: The swelling-inhibitory effect of 100 µM triamcinolone acetonide (Triam) was abrogated by 100 nM DPCPX, a A_1_ receptor blocker. **C**: The cell-permeable calcium chelator BAPTA/AM (100 µM) did not reverse the swelling-inhibitory action of 10 µM adenosine. The swelling of Müller cells was abrogated in the presence of a cAMP-enhancing cocktail containing 100 µM pCPT-cAMP, 10 µM forskolin, and 100 µM IBMX. **D**: The effect of 10 µM adenosine was prevented in the presence of 10 µM clofilium, a potassium channel blocker, and 100 µM NPPB, a chloride channel blocker. Data are mean (±SEM) soma areas (n=7–12 cells per bar). Significant differences versus control (the asterisk indicates a p<0.001). Significant swelling-inhibitory effects (the solid circle indicates a p<0.001). Significant inhibition of the agonist effects (the open circle indicates a p<0.001).

It has been suggested that triamcinolone acetonide prevents the osmotic swelling of rat Müller cells via activation of adenosine receptors [20]. We found that exogenous adenosine inhibited the osmotic swelling of Müller cells in retinal slices from wild-type mice. This effect was abrogated by a selective blocker of A_1_ receptors (Figure 2A). Triamcinolone acetonide prevented the osmotic swelling of Müller cells, and this result was also abrogated in the presence of the A_1_ receptor blocker (Figure 2B). The data suggest that triamcinolone evokes a release or generation of endogenous adenosine and subsequent activation of A_1_ receptors.

To determine the intracellular signaling which is evoked by adenosine, we tested the cell-permeable calcium chelator BAPTA/AM. As shown in Figure 2C, calcium chelation did not abrogate the inhibitory action of adenosine on the osmotic swelling of Müller cells, disclosing the possibility that the effect of adenosine is mediated by intracellular calcium signaling. cAMP was suggested to be a downstream intracellular mediator of adenosine action in Müller cells of the rat [30,31]. We found that the osmotic swelling of Müller cells from wild-type mice was abrogated in the presence of a cAMP-enhancing cocktail (Figure 2C). Prevention of osmotic cell swelling can be achieved by opening of ion channels in the plasma membrane; the transmembrane ion flux compensates the osmotic gradient across the membrane and, thus, prevents cellular swelling. We found that the swelling-inhibitory effect of adenosine was absent in the presence of the class III antiarrhythmic drug, clofilium, and the blocker of chloride channels, NPPB, respectively (Figure 2D). Clofilium is a known blocker of two pore-domain potassium channels [32] which have been shown to be expressed by Müller cells [33]. The data suggest that stimulation of A_1_ receptors leads to activation of “background” potassium channels, as well as chloride channels, that mediate the ion efflux required for the prevention of Müller cell swelling.

**Figure 3:**
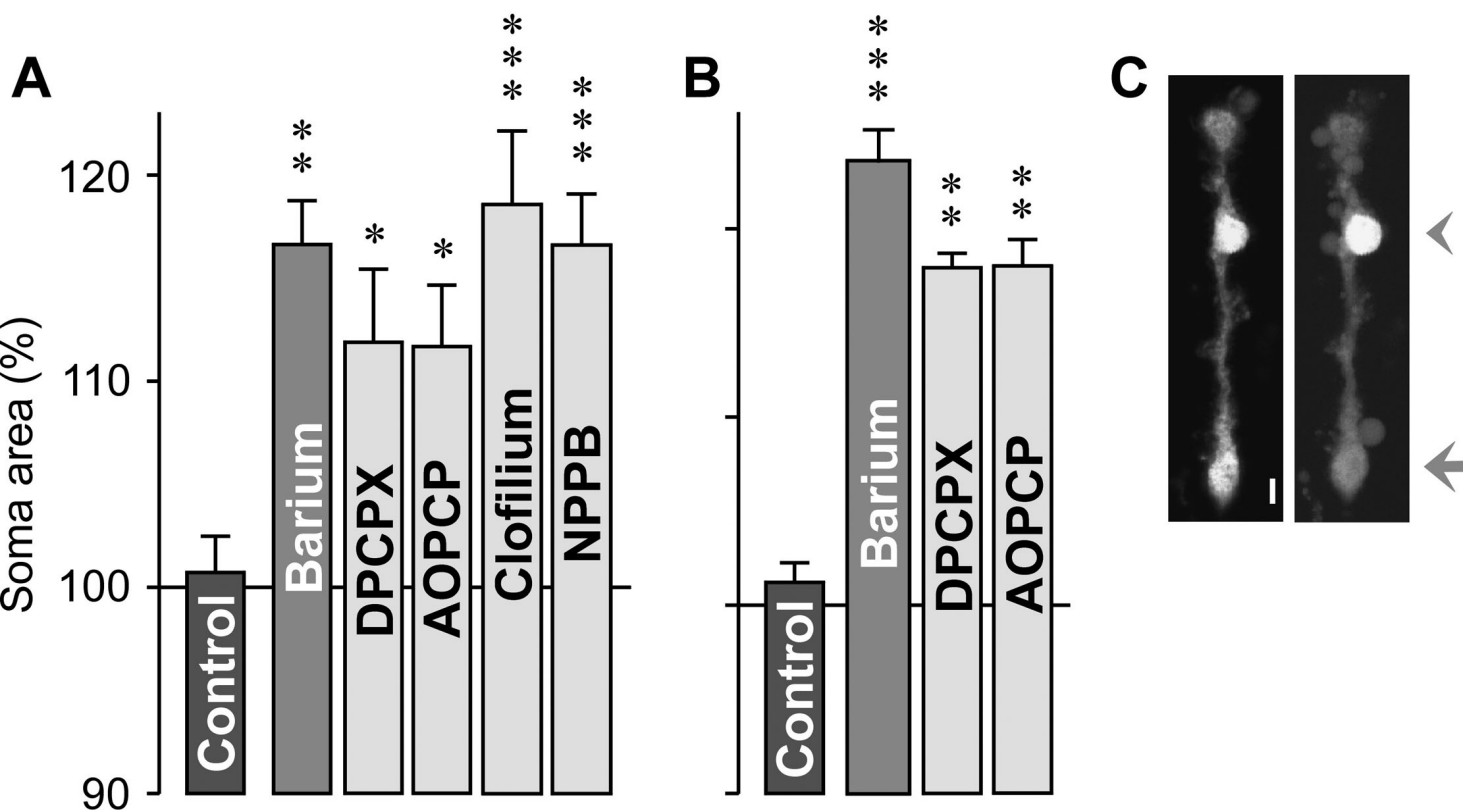
Endogenous adenosine signaling is required to prevent the osmotic swelling of Müller cells. Data were obtained in freshly isolated retinal slices (**A**) and Müller cells (**B**) from wild-type mice. Perfusion of the slices or cells with a hypoosmolar solution containing 1 mM barium chloride, 100 nM DPCPX, a A_1_ receptor blocker, 100 µM AOPCP, an ecto-5′-nucleotidase inhibitor, 10 µM clofilium, a potassium channel blocker, or 100 µM NPPB, a chloride channel blocker, resulted in a swelling of Müller cell somata. **C**: Original records of a dye-filled isolated Müller cell before (left) and during (right) perfusion with the barium-containing hypoosmolar solution. The arrowhead indicates the cell soma, and the arrow refers to the cell endfoot. The scale bar equals 5 µm. Data are mean (±SEM) soma areas (n=7–14 cells per bar) which were measured after a 4 min perfusion of the hypoosmolar solution. Data are expressed in percent of the soma size measured before hypotonic challenge (100%). Significant differences versus control (the asterisk indicates a p<0.05, the double asterisk indicates a p<0.01, and the triple asterisk indicates a p<0.001).

We found that Müller cells in freshly isolated retinal slices from wild-type mice did not significantly swell within 4 min of hypotonic exposure (Figure 1B). To reveal whether endogenous adenosine signaling is required for this volume homeostasis of Müller cells, we recorded the osmotic swelling of Müller cells in retinal slices from wild-type animals in the presence of different pharmacological antagonists. As shown in Figure 3A, the presence of a selective inhibitor of A_1_ receptors resulted in a swelling of the Müller cell bodies under hypoosmotic conditions which was not observed in the absence of the blocking agent. In addition, pharmacological inhibition of the ecto-5′-nucleotidase (CD73) resulted in osmotic swelling of Müller cells (Figure 3A). CD73 is known to hydrolyze nucleoside monophosphates such as AMP to the respective nucleosides [34]; Müller cells are known to express CD73 [35,36]. The amplitude of soma swelling in the presence of the blocking agents was smaller than the amplitude of the barium-evoked swelling (Figure 3A). The data may suggest that endogenous adenosine signaling (involving extracellular generation of adenosine and activation of A_1_ receptors) is required to prevent osmotic swelling of Müller cells under hypoosmotic conditions. The swelling-inducing effects of the potassium and chloride channel blockers, respectively, clofilium and NPPB (Figure 3A), suggest that hypoosmotic swelling of Müller cells is normally prevented by opening of ion channels in the plasma membrane.

To reveal whether the endogenous adenosine signaling is evoked by generation of adenosine at the surface of Müller cells or retinal neurons, we tested the pharmacological blockers in freshly isolated Müller cells from wild-type mice. As shown in Figure 3B, hypotonic challenge evoked a swelling of Müller cell bodies in the presence (but not absence) of selective inhibitors of A_1_ receptors and CD73, respectively. The data suggest that adenosine is generated at the surface of Müller cells in response to hypoosmotic stress.

### Osmotic swelling of Müller cells from A_1_AR^−/−^ mice

**Figure 4:**
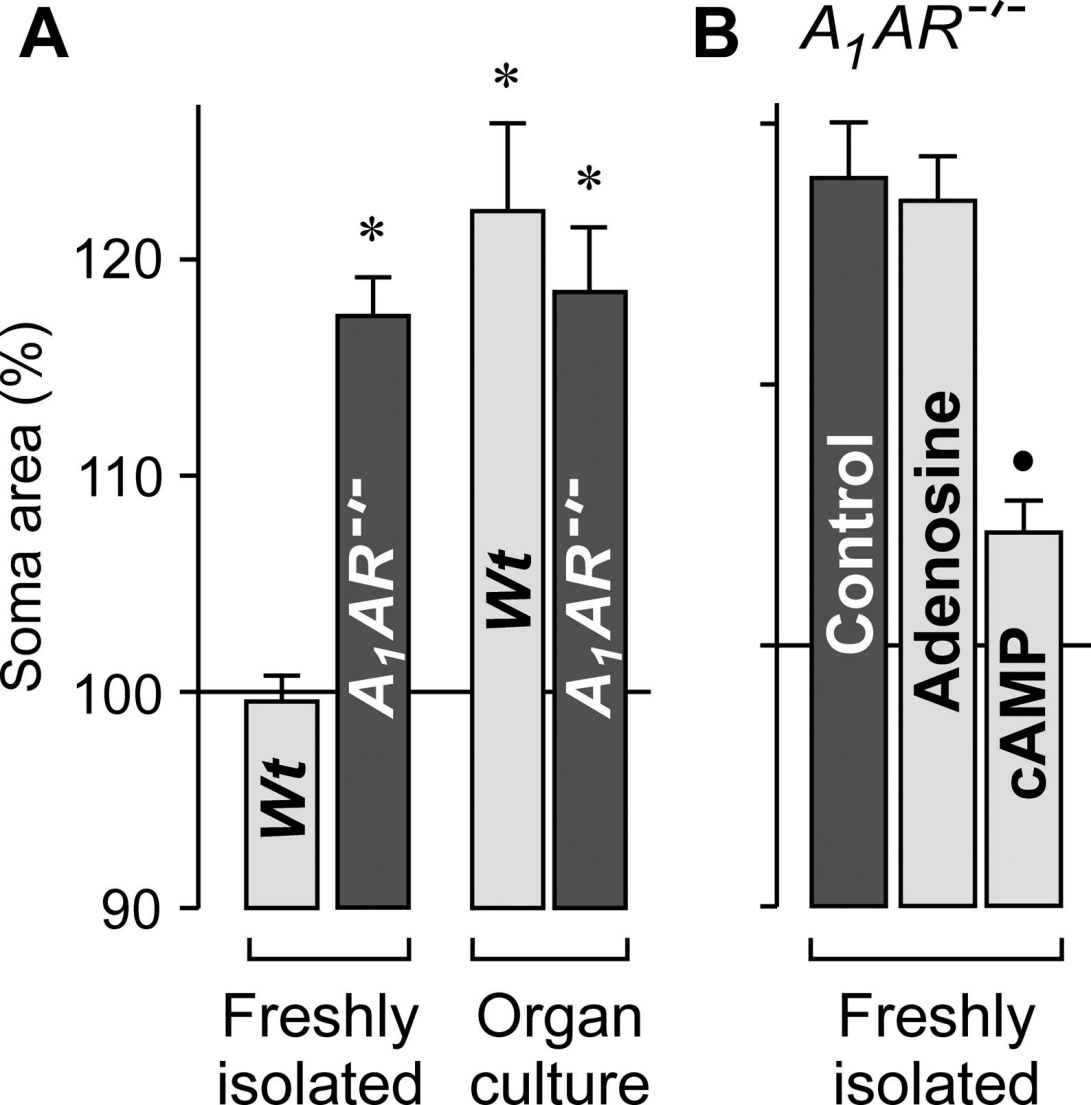
Osmotic swelling properties of Müller cells from *A_1_AR^−/−^* and wildtype (*Wt*) mice. The cross-sectional area of Müller cell somata was measured after a 4 min perfusion of retinal slices with a hypoosmolar solution (in the absence of barium), and are expressed in percent of the soma size measured before hypotonic challenge (100%). **A**: Hypoosmotic exposure evoked swelling of Müller cell bodies in retinal slices from *A_1_AR^−/−^* mice. This was observed in freshly isolated retinas and retinal organ cultures. In contrast, in tissues from wild-type animals, swelling was induced in retinal organ cultures but not in freshly isolated retinas. **B**: Müller cells in retinal slices derived from *A_1_AR^−/−^* mice displayed a swelling of their cell bodies under hypoosmotic conditions (control). In slices of these mice, 10 µM adenosine did not prevent the swelling of the cells. The swelling was diminished in the presence of a cAMP-enhancing cocktail containing 100 µM pCPT-cAMP, 10 µM forskolin, and 100 µM IBMX. Significant differences versus freshly isolated cells from wild-type mice (the asterisk indicates a p<0.001). Significant swelling-inhibitory effect (the filled circle indicates a p<0.001).

The aforedescribed pharmacological data suggest that endogenous adenosine signaling via A_1_ receptors is required to prevent osmotic Müller cell swelling. To prove this assumption, we compared the swelling characteristics of Müller cells in retinal slices from wild-type and A_1_ adenosine receptor-deficient mice. As shown in Figure 4A, perfusion of freshly isolated retinal slices from *A_1_AR^−/−^* mice with a hypoosmolar solution (without barium) for 4 min evoked a significant (p<0.001) swelling of Müller cell bodies which was not observed in freshly isolated slices from wild-type mice. However, Müller cells in retinal organ cultures from both wild-type and *A_1_AR^−/−^* mice displayed cellular swelling under hypoosmotic conditions (Figure 4A). The data suggest that Müller cells of *A_1_AR^−/−^* mice are disturbed in their cell volume regulation in response to osmotic stress, likely due to a disruption of the endogenous adenosine signaling which is normally involved in the regulation of Müller cell volume. In freshly isolated retinal slices from *A_1_AR^−/−^* mice, adenosine failed to inhibit the osmotic swelling of Müller cells (Figure 4B), suggesting that other subtypes of adenosine receptors are not involved in the regulation of Müller cell volume. Yet, a cAMP-enhancing cocktail diminished the osmotic swelling of Müller cells from *A_1_AR^−/−^* mice (Figure 4B).

### Electrophysiological and immunohistochemical characterization of Müller cells

**Figure 5:**
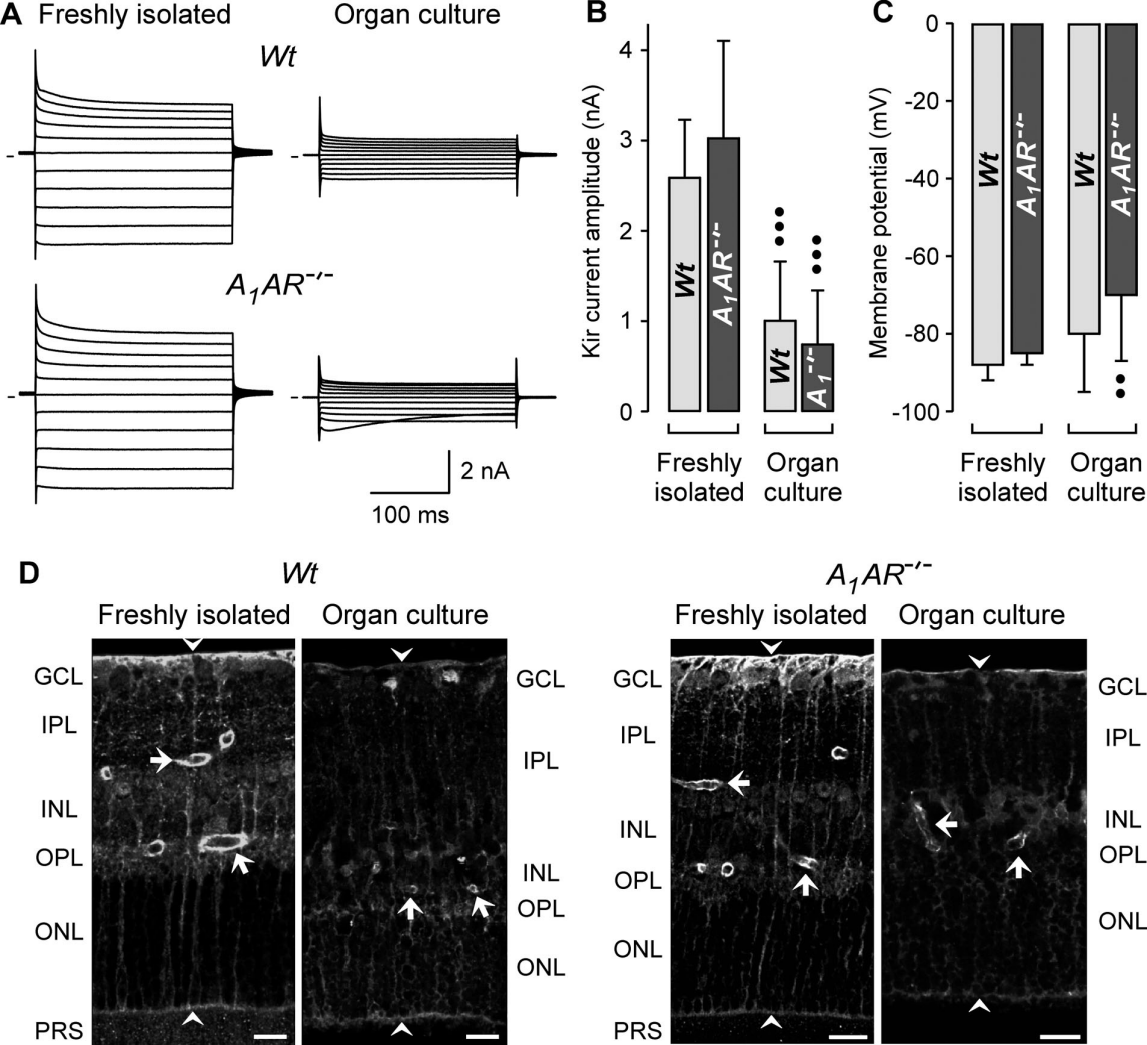
Organ culturing alters the potassium conductance of Müller glial cells and the retinal immunolabeling of Kir4.1 protein. Retinal tissues from wild-type (*Wt*) and *A_1_AR^−/−^* mice were used. The tissues were freshly isolated or derived from retinal organ cultures. **A**: Shown are examples of original records of whole-cell potassium currents, which were obtained in isolated Müller cells. Outward (upwardly depicted) and inward (downwardly depicted) currents were evoked by 20 mV incremental voltage steps up to +20 and −180 mV from a holding potential of −80 mV. The lines at left of each trace indicate zero current levels. **B**: Retinal organ culturing results in a decrease of the mean amplitude of the Kir currents of Müller cells. **C**: Retinal organ culturing results in a slight decrease in the mean resting membrane potential of Müller cells from *A_1_AR^−/−^* mice. **D**: Retinal organ culturing results in a decrease in the intensity of the Kir4.1 immunoreactivity. The arrows indicate perivascular staining, and the arrowheads point to the limiting membranes of the retina. Abbreviations: ganglion cell layer (GCL); inner nuclear layer (INL); inner plexiform layer (IPL); outer nuclear layer (ONL); outer plexiform layer (OPL); photoreceptor segments (PRS). Scale bars equal to 20 µm. The bar diagrams display mean (±SD) values obtained in 6–22 cells. Significant differences versus the respective control obtained in freshly isolated cells (the double closed circles indicate a p<0.01 and the triple closed circles indicate a p<0.001).

We found that Kir channel-blocking barium ions induced a swelling of Müller cell bodies in freshly isolated retinal slices of wild-type mice (Figures 1A,B). Because Müller cells in slices from *A_1_AR^−/−^* mice displayed an alteration in the osmotic swelling characteristics when compared to cells from wild-type mice, we determined whether this alteration was associated with a decrease in the Kir currents of the cells. As shown in Figure 5A, freshly isolated Müller cells of both wild-type and *A_1_AR^−/−^* mice displayed large potassium currents around the resting membrane potential (at the zero current level) and upon membrane hyperpolarization. The mean amplitude of the Kir currents (Figure 5B) and the resting membrane potential (Figure 5C) were not different between freshly isolated cells from both strains. Furthermore, the mean membrane capacitance which is proportional to the plasma membrane area of the cells did not differ between freshly isolated cells from both mouse strains (data not shown), disclosing the possibility of cellular hypertrophy of cells from the knockout mice.

To reveal whether deficiency in A_1_ receptors may result in retinal gliosis, we immunostained freshly isolated retinal slices from both mouse strains against the glial intermediate filament GFAP and the major glial Kir channel subtype (Kir4.1). Alterations in the retinal distribution of Kir4.1 protein have been described to occur in rat models of various retinopathies [21-23,37]. We found that the Kir4.1 protein displayed similar distributions in freshly isolated retinal slices from wild-type and *A_1_AR^−/−^* mice, with enrichments at both limiting membranes and around the blood vessels (Figure 5D). Upregulation of GFAP is a common marker of Müller cell gliosis [37]. We found in slices from both mouse strains that the GFAP labeling was restricted to the innermost retinal layers, i.e., nerve fiber and ganglion cell layers (not shown), suggesting that GFAP was largely expressed by retinal astrocytes but not Müller cells. The unaltered potassium conductance and plasma membrane area of Müller cells, and the similar retinal distributions of GFAP and Kir4.1 proteins, largely rule out the possibility that deficiency of A_1_ receptors results in Müller cell gliosis under normal conditions.

Under culture conditions, Müller cells from both animal strains displayed a similar decrease in the amplitude of their Kir currents when compared to freshly isolated cells (Figures 5A,B). This was associated with a slight membrane depolarization in the case of cells from *A_1_AR^−/−^* mice (Figure 5C). Though the overall distribution of the Kir4.1 protein in retinal slices did not alter under culture conditions, the staining level decreased in retinal slices from organ cultures compared to freshly isolated retinal slices (Figure 5D). The decrease in the staining level was obvious in retinal slices from both wild-type and *A_1_AR^−/−^* animals.

## Discussion

**Figure 6:**
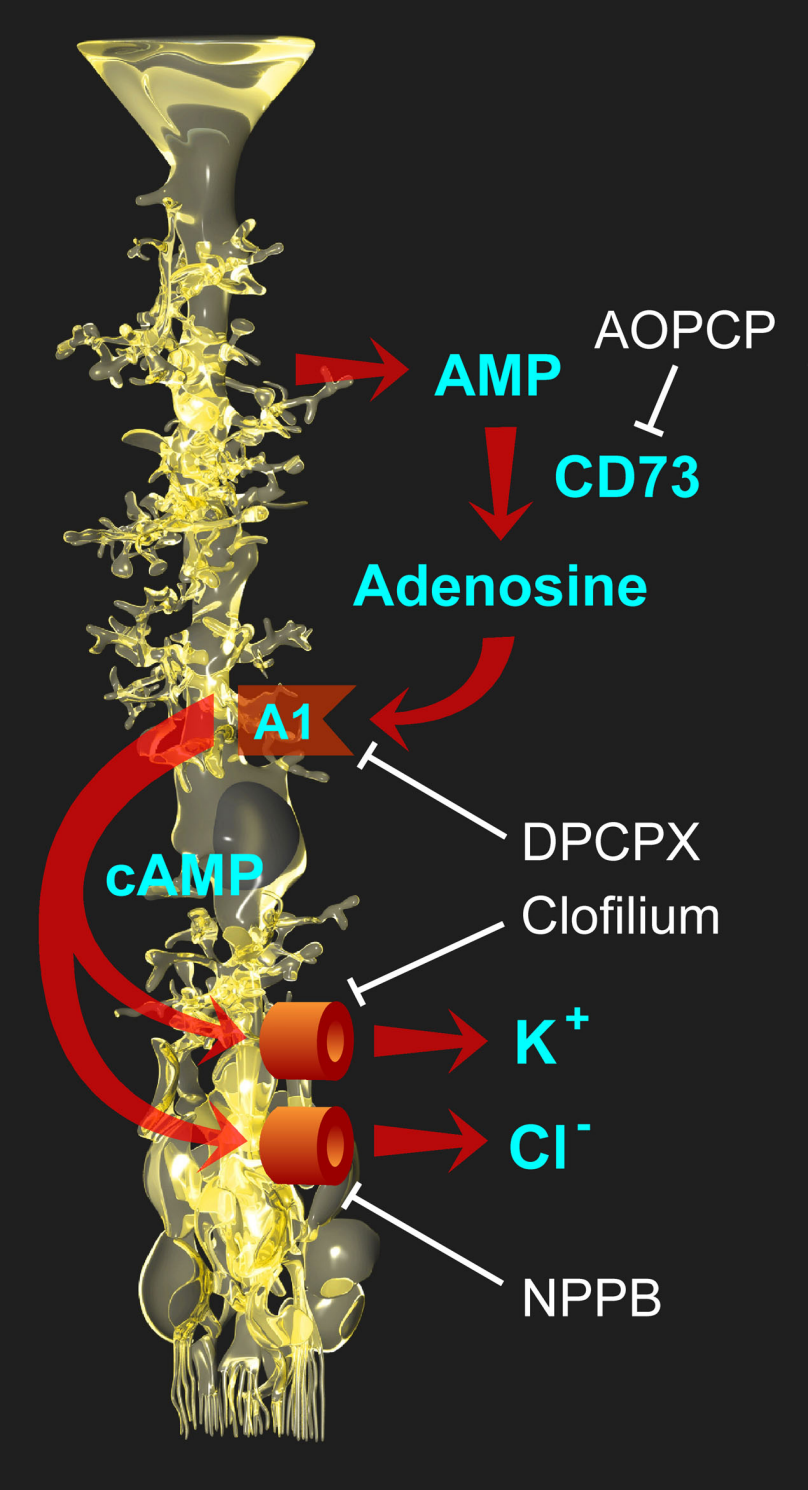
Involvement of adenosine signaling in the endogenous osmotic volume regulation of retinal glial cells in mice. Adenosine, which is extracellularly formed from AMP by the action of CD73, activates A_1_ receptors of Müller cells. Activation of the receptors triggers (via intracellular formation of cAMP) the opening of potassium and chloride channels in the Müller cell membrane. The ion efflux from the cells equalizes the osmotic gradient across the plasma membrane and thus prevents cellular swelling under hypotonic conditions. Different steps of the signaling cascade can be blocked by specific antagonists (shown in white), resulting in cellular swelling when the extracellular osmolarity decreases.

Regulation of the Müller cell volume has great importance for the homeostasis of the extracellular space volume under conditions of intense neuronal activity. It has been shown that activation of neuronal ionotropic glutamate receptors causes a net uptake of sodium chloride, resulting in a swelling of neuronal cell bodies and synapses, and a decrease in the extracellular space volume [38]. Light-evoked changes in the ionic composition of the extracellular space fluid, with a decrease in sodium which is about twice as large as the increase in potassium, cause a decrease in the osmolarity of the extracellular fluid [39]. The uptake of neuron-derived osmolytes, such as potassium and sodium glutamate [37,40], may further enhance the osmotic gradient across Müller cell membranes. To avoid an excessive decrease in the extracellular space volume, which may result in neuronal hyperexcitability [41,42], Müller cells must maintain their volume constant, or decrease their volume, when neuronal cell structures swell and the extracellular osmolarity decreases during neuronal activity. The present results suggest that, in the murine retina, an endogenous adenosine signaling mediates the volume homeostasis of Müller cells in response to varying osmotic conditions. A disruption of this signaling by pharmacological inhibition of A_1_ receptors or targeted deletion of A_1_ receptors results in a swelling of Müller cells when the environment is hypoosmolar in comparison to the Müller cell interior. The present data suggest that CD73-dependent extracellular production of adenosine, activation of A_1_ receptors, an increase in cytosolic cAMP, and an opening of potassium and chloride channels are events in mediating the volume homeostasis of Müller cells in the mouse retina (Figure 6). The swelling of Müller cells from *A_1_AR^−/−^* mice (Figure 4A) and of wild-type cells during pharmacological blockade of A_1_ receptors (Figures 3A,B), and the absence of a swelling-inhibitory effect of adenosine in cells from *A_1_AR^−/−^* mice (Figure 4B), suggest that other receptor systems cannot compensate the missing action of A_1_ receptors in the regulation of Müller cell volume.

We found that pharmacological blockade of A_1_ receptors or inhibition of the activity of the ecto-5′-nucleotidase resulted in a swelling of Müller cells with an amplitude slightly smaller than the amplitude of the barium-evoked swelling (Figure 3A). Therefore, it cannot be ruled out that, in addition to the A_1_ receptor-mediated mechanism, other mechanisms contribute to the volume regulation of Müller cells. One of these mechanisms may be the passive efflux of potassium ions through Kir channels [8,21]. We found that culturing of retinal tissues results in an impairment of the rapid volume regulation of Müller cells (Figure 1B), and is associated with a decrease in the Kir currents of Müller cells (Figures 5A,B) and an apparent decrease in the retinal level of Kir4.1 protein (Figure 5D). A decrease in the expression of functional Kir channels simultaneously with an induction of osmotic swelling of Müller cells were found to be characteristic events in animal models of various retinopathies, including retinal ischemia and inflammation [21,22]. However, because Müller cells from both wild-type and *A_1_AR^−/−^* mice have similar Kir currents (Figures 5A,B) but different swelling characteristics (Figure 4A), the functional role of Kir channels in preventing Müller cell swelling remains to be determined. It seems to be likely (but remains to be proven) that a disruption of the endogenous adenosine signaling normally involved in the regulation of Müller cell volume is involved in the induction of Müller cell swelling under pathological conditions. The absence of retinal gliosis and degeneration in *A_1_AR^−/−^* mice may suggest that alternative mechanisms of (less efficient but still sufficient) glial volume homeostasis, as well as a low level of neuronal activity, may compensate for the deficit—and maintain retinal integrity—in these mice. It could be that in retinas of these mice, other (presently undefined) cAMP-enhancing factors beside adenosine may play a volume-regulatory role.

Apparently, the swelling-inhibitory effect of A_1_ receptor activation is mediated by the opening of barium-insensitive potassium channels (likely two pore-domain channels [33]), as well as of chloride channels, in the Müller cell membrane. The efflux of ions balances the osmotic gradient across the plasma membrane and thus prevents cellular swelling under hypoosmotic conditions. In swollen cells, the ion efflux is associated with an efflux of water from the cells which results in a decrease of the cell volume. Two pore-domain potassium channels may function as an osmolyte extrusion pathway that helps to maintain a proper Müller cell volume when Kir4.1 channels are downregulated or inactivated under pathological conditions. Under normal conditions, these channels may regulate the osmotic balance of such Müller cell regions that are largely devoid of Kir4.1, including the somata, which were recorded in the present study.

Triamcinolone acetonide is used clinically for the rapid resolution of retinal edema [11,12]. Based upon findings in the human retina [5,6] and in rat models of retinopathies [21-24], it has been suggested that osmotic swelling of Müller cells may contribute to the development of retinal edema [7,8]. In rat retinal tissues, triamcinolone has been shown to prevent osmotic swelling of Müller cells [20]. Here, we show that triamcinolone inhibits the osmotic swelling of Müller cells in the murine retina (Figure 2B). The inhibitory effect of an A_1_ receptor blocker on the action of triamcinolone (Figure 2B) suggests that the swelling-inhibitory effect of this steroid is mediated by stimulation of the endogenous adenosine signaling. Adenosine, which is rapidly released in the retina upon ischemia or hypoxia [43,44], is an important component of the retinal response to ischemic-hypoxic stress. Activation of A_1_ receptors has a protective effect against ischemic injury of the retina [45,46]. It remains to be determined whether the neuroprotective effect of triamcinolone found in an animal model of subretinal hemorrhage [47] is, at least in part, mediated by stimulation of the endogenous adenosine signaling. It has been suggested that potassium currents drive the activity-dependent water transport through Müller cells [7]. Thus, activation of two pore-domain channels by triamcinolone may facilitate the potassium clearance and the water absorption from the edematous retinal tissue. A stimulatory effect on the fluid clearance through Müller cells may contribute to the rapid edema-resolving effect of triamcinolone.
